# *N*-Acetylcysteine Alleviated the Deltamethrin-Induced Oxidative Cascade and Apoptosis in Liver and Kidney Tissues

**DOI:** 10.3390/ijerph19020638

**Published:** 2022-01-06

**Authors:** Ali Allam, Ahmed Abdeen, Hari Prasad Devkota, Samar S. Ibrahim, Gehan Youssef, Ahmed Soliman, Mohamed M. Abdel-Daim, Khalid J. Alzahrani, Khaled Shoghy, Samah F. Ibrahim, Mohamed Aboubakr

**Affiliations:** 1 Department of Pharmacology, Faculty of Veterinary Medicine, Benha University, Toukh 13736, Egypt; alitahaallam@gmail.com (A.A.); mohamed.aboubakr@fvtm.bu.edu.eg (M.A.); 2 Department of Forensic Medicine and Toxicology, Faculty of Veterinary Medicine, Benha University, Toukh 13736, Egypt; samar.mohamed@fvtm.bu.edu.eg (S.S.I.); gihan.basiony@fvtm.bu.edu.eg (G.Y.); 3 Center of Excellence in Screening of Environmental Contaminants (CESEC), Benha University, Toukh 13736, Egypt; 4 Graduate School of Pharmaceutical Sciences, Kumamoto University, 5-1 Oe-Honmachi, Chuo-ku, Kumamoto 862-0973, Japan; 5 Pharmacology Department, Faculty of Veterinary Medicine, Cairo University, Giza 12211, Egypt; galalpharma@cu.edu.eg; 6 Department of Pharmaceutical Sciences, Batterjee Medical College, P.O. Box 6231, Jeddah 21442, Saudi Arabia; abdeldaim.m@vet.suez.edu.eg; 7 Pharmacology Department, Faculty of Veterinary Medicine, Suez Canal University, Ismailia 41522, Egypt; 8 Department of Clinical Laboratories Sciences, College of Applied Medical Sciences, Taif University, P.O. Box 11099, Taif 21944, Saudi Arabia; ak.jamaan@tu.edu.sa; 9 Department of Anatomy and Embryology, Faculty of Veterinary Medicine, University of Sadat City, Sadat City 32897, Egypt; khaled.shoghy@vet.usc.edu.eg; 10 Department of Clinical Sciences, College of Medicine, Princess Nourah Bint Abdulrahman University, P.O. Box 84428, Riyadh 11671, Saudi Arabia; sfibrahim@pnu.edu.sa

**Keywords:** deltamethrin, *N*-acetylecystine, caspase3, Bcl2, hepato-renal toxicity, antioxidant

## Abstract

Deltamethrin (DLM) is a synthetic pyrethroid with anti-acaricide and insecticidal properties. It is commonly used in agriculture and veterinary medicine. Humans and animals are exposed to DLM through the ingestion of polluted food and water, resulting in severe health issues. *N*-acetylcysteine (NAC) is a prodrug of L-cysteine, the precursor to glutathione. It can restore the oxidant-antioxidant balance. Therefore, this research aimed to examine whether NAC may protect broiler chickens against oxidative stress, at the level of biochemical and molecular alterations caused by DLM intoxication. The indicators of liver and kidney injury in the serum of DLM-intoxicated and NAC-treated groups were examined. Furthermore, lipid peroxidation, antioxidant markers, superoxide dismutase activity, and apoptotic gene expressions (caspase-3 and Bcl-2) were investigated. All parameters were significantly altered in the DLM-intoxicated group, suggesting that DLM could induce oxidative damage and apoptosis in hepato-renal tissue. The majority of the changes in the studied parameters were reversed when NAC therapy was used. In conclusion, by virtue of its antioxidant and antiapoptotic properties, NAC enabled the provision of significant protection effects against DLM-induced hepato-renal injury.

## 1. Introduction

**Figure 1:**
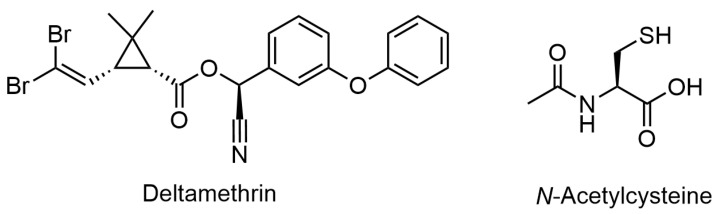
Chemical structures of deltamethrin and *N*-acetylcysteine.

Insecticides have been used in agriculture for a long time and have a significant impact on rising agricultural production [1]. Since the sale of organophosphorus insecticides is now ruled by stricter government regulations, pyrethroids have emerged as the preferred insecticide, and their use has skyrocketed [2]. Deltamethrin (DLM; Figure 1) is an essential member of the pyrethroid family and is considered a type II pyrethroid. Among organophosphorus pesticides, DLM is the most widely used pesticide. DLM is an extensively used insecticide in many agricultural and veterinary applications to combat insects, including controlling insects in poultry farms [3]. Therefore, DLM is considered as a potential environmental contaminant that poses health risks to humans and a variety of animals [4,5]. Direct inhalation of DLM spray during application or ingestion of DLM-contaminated food and drink are the most common avenues of exposure [6]. It has the ability to, directly and indirectly, disrupt the DNA structure and cellular antioxidant competence [7]. Hepatotoxicity, nephrotoxicity, genotoxicity, immunosuppression, mutagenicity, and infertility are common health hazards of DLM toxicity [8].

Depletion of the endogenous antioxidant system along with the overproduction of reactive oxygen species (ROS) are the main mechanisms involved in the DLM-induced oxidative damage and consequently tissue injury [9,10]. DLM is largely excreted by the kidney and is metabolized by the hepatic microsomal enzyme system, intestinal esterases, and plasma carboxylesterases [11]. These metabolites can accumulate in the hepatic and renal tissue due to the initiation of oxidative damage [12]. In addition to conventional DLM poisoning therapies, antioxidants may be promising agents for scavenging ROS and preserving the antioxidant enzyme activities [13,14]. DLM can cause apoptosis in cells by activating mitochondria-mediated apoptosis [15].

*N*-acetylcysteine (NAC; Figure 1) is an amino acid that contains a thiol group. In the 1960s, it was available as a safe and inexpensive mucolytic drug [16]. Despite the fact that cysteine occurs naturally in a variety of foods, NAC is not a natural drug, but it is a precursor of the amino acid L-cysteine. It is well known that L-cysteine is essential for the synthesis and regeneration of reduced-glutathione (GSH). As a one of the most effective antioxidants compounds, GSH has the ability to protect tissues from the harmful effects of ROS under oxidative stress conditions [17]. Therefore, NAC is a promising therapy for a number of xenobiotic-induced oxidative stress [14]. It has been used to treat heart injury, lung disease, and cancer for many decades [18]. Dietary NAC has been shown to reduce liver damage in a porcine model by enhancing the cellular antioxidant ability and energy metabolism [19]. Moreover, in a study conducted by Yi et al. [20], NAC has greatly improved the cellular antioxidant potency building up a protective panel against aflatoxicosis in broiler chickens.

Thus, the aim of this study was to evaluate whether NAC could protect broiler chickens against DLM-induced toxicity. Body weight gain, feed conversion rate, serum biochemistry, tissue lipid peroxidation, antioxidant biomarkers, and caspase3 and Bcl2 mRNA expression were determined in DLM- and/or NAC-treated broiler chickens.

## 2. Materials and Methods

### 2.1. Chemicals

Deltamethrin (Butox^®^, 50 mg/mL) was bought as a commercial product in clinical formulation for veterinary use from Intervet Co. (Paris, France). While, *N*-acetyl-l-cysteine was purchased as a commercial product from the South Egypt Drug Industries Company, SEDICO (6th of October, SEDICO, Cairo, Egypt).

### 2.2. Experimental Animals

One-day-old, apparently healthy, Cobb broiler chicks were purchased from the El-Wataniya Poultry Company (Egypt). Chicks were divided into four experimental groups; each was performed into three replicates of five chicks:

- The first group (control) received a basal diet without any treatments;
- The second group (NAC) fed on a diet supplemented with NAC, 5.2 g/kg diet [21];
- The third group (DLM) fed on a diet contaminated with DLM 300 mg/kg diet [22];
- The fourth group (DLM+NAC) fed on a diet mixed with DLM and NAC, at the same rates of the above-mentioned doses.

Different treatments were given to all of the groups for 35 days. The chicks were kept in separate units on the floor for sanitary reasons. Per week, the starting temperature of 32 °C was reduced by 2 °C. Continuous lighting was used, and food and water were freely available. Diet was formulated as starter and finisher to meet the nutritional requirements (Table 1). The chicks were vaccinated according to the local immunization program’s guidelines (Newcastle disease and infectious bronchitis viruses). The experiment was conducted and approved according to the guidelines of the Ethical Committee of the Faculty of Veterinary Medicine, Benha University (Approval number; BUFVTM 06-03-21).

### 2.3. Growth Parameters

The feed intake and weight gain were measured daily. Feed conversion ratio (FCR) was measured at the completion of the experiment according to the following formula.

(1) $$\mathrm{FCR}=\frac{Feed\;consumption}{Body\;weight\;gain}$$

### 2.4. Blood Samples

At the end of the experiment, blood samples were collected from each group through wing vein and at room temperature for clotting. Clear sera were harvested by centrifugation at 2000× *g* for 10 min, then carefully transferred into clean dry vials and frozen at −20 °C until further biochemical examinations.

### 2.5. Hepato-Renal Function Tests

Liver and kidney function tests including alanine aminotransferase (ALT), aspartate aminotransferase (AST), creatinine, and urea were estimated using commercially available kits according to the manufacturer’s instructions (Laboratory Biodiagnostics, Giza, Egypt).

### 2.6. Determination of Oxidative Stress Markers

After dissection of liver and kidney, red blood cells and clots were removed by perfusion in a phosphate buffered saline solution (pH 7.4) containing 0.16 mg/mL heparin. One gram of each tissue was homogenized in 5 mL of cold buffer (50 mM potassium phosphate and 1 mM EDTA, pH 7.5). Aliquots of tissue homogenates were centrifuged at 4000 rpm/20 min/4 °C and then kept at −20 °C until use. Malondialdehyde (MDA) and GSH levels and superoxide dismutase (SOD) activity were measured using commercially available kits (Laboratory Biodiagnostics, Giza, Egypt).

### 2.7. Real-Time PCR

The total RNA was extracted from liver and kidney tissues using RNeasy mini kit (Qiagen, Germantown, MD, USA). Then, cDNA was synthesized using a high capacity cDNA reverse transcription kit (Qiagen, USA). The transcription levels of the apoptotic genes (caspase3 and Bcl2) were normalized to the housekeeping gene β-actin. The mRNA expression level was quantified using the standard 2^−ΔΔCT^ method [23]. The specific primer sequences of caspase3, Bcl2, and β-actin were designed as reported previously and are presented in Table 2.

### 2.8. Examination of Histopathology

Specimens from the liver and kidney were taken from slaughtered chickens and fixed for at least 24 h in a 10% formalin solution. Thereafter, all samples were dehydrated in graded dilutions of ethanol followed by paraffin embedding. Next, the obtained paraffin blocks were sectioned into 4 µm thickness and stained with hematoxylin and eosin (H&E).

### 2.9. Statistical Analysis

Using SPSS version 20.0 software, the variations between the averages were calculated using the analysis of variance test (one-way ANOVA) and Duncan’s multiple range tests (SPSS Inc., Chicago, IL, USA). All data is provided in average ± standard error (SE).

## 3. Results

### 3.1. Effect of DLM and/or NAC on Growth Performance

As presented in Table 3, DLM treatment group resulted in significant decreases in the body weight, weight gain, and FCR of broiler chickens. Meanwhile, the NAC-treated group gained weight and had a lower FCR compared to the control chickens. The DLM+NAC-treated group showed an improvement in the growth performance in comparison with the DLM-treated group.

### 3.2. The Effect of DLM and/or NAC on Biochemical Markers of the Liver and Kidney

**Figure 2:**
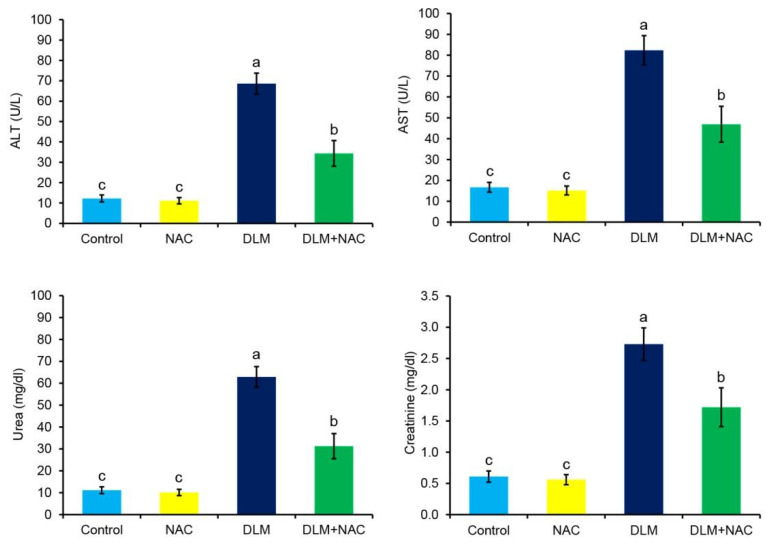
Effect of DLM and/or NAC treatments on the hepato-renal function. The values are expressed using the mean ± SE (*n* = 3). The statistically significant difference (*p* ≤ 0.05) is indicated by different superscript characters.

In comparison to the control group, there are significant increases in serum ALT, AST, urea, and creatinine in response to DLM treatment. On the other hand, serum from the NAC treated group showed no significant changes in such markers (Figure 2). However, when DLM-intoxicated chickens were simultaneously treated with NAC, marked restoration of liver and kidney function tests were observed (Figure 2).

### 3.3. The Effect of DLM and/or NAC on Oxidative Cascade in Liver and Kidney Tissues

**Figure 3:**
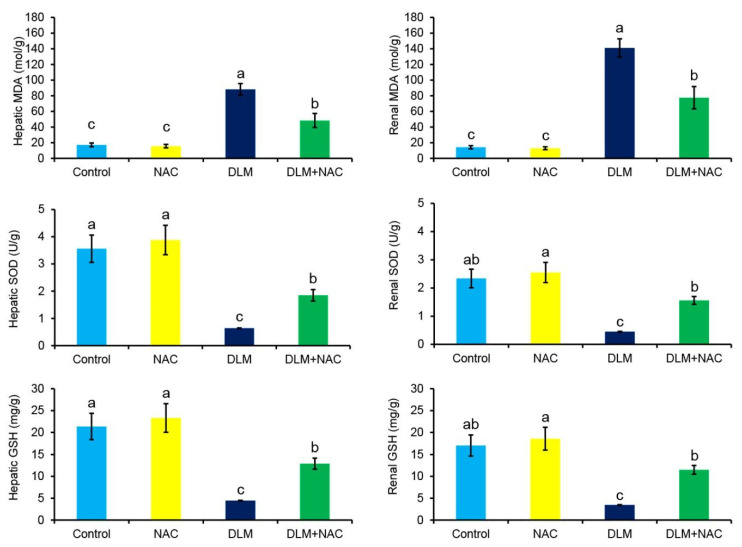
Effect of DLM and/or NAC treatments on hepatic and renal oxidative indexes. The values are expressed using the mean ± SE (*n* = 3). The statistically significant difference (*p* ≤ 0.05) is indicated by different superscript characters.

As depicted in Figure 3, the control and NAC groups exhibited no alterations in the oxidative stress indexes. However, homogenate of the chicken’s liver or kidney tissues of DLM-treated birds showed a dramatic increase in MDA level and a significant decrease in SOD activity and GSH concentration compared to controls. Notably, administration of NAC along with DLM could markedly alleviate the DLM-induced alterations in the oxidative stress markers when compared to DLM group.

### 3.4. The Effect of DLM and/or NAC on Caspase-3 and Bcl2 mRNA Expression in Liver and Kidney Tissues

**Figure 4:**
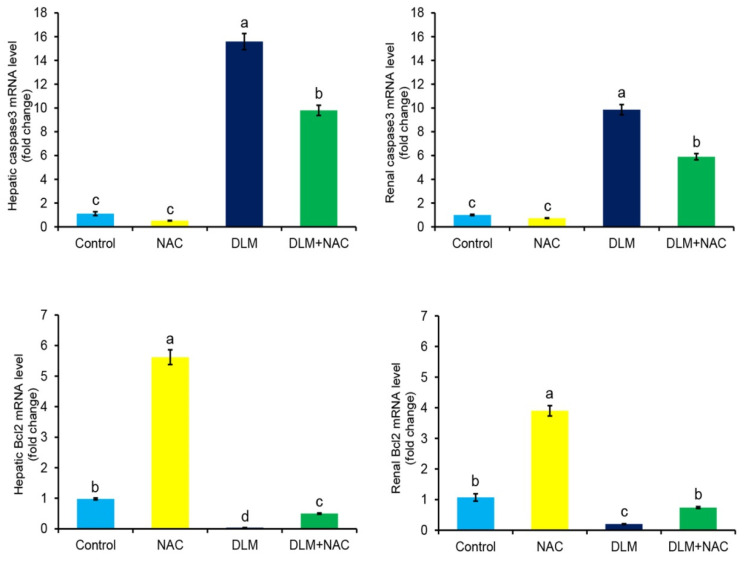
Effect of DLM and/or NAC treatments on caspase3 and Bcl2 mRNA expression levels. The values are expressed using the fold changes ± SE (*n* = 3), after normalization against the endogenous control (β-actin). The statistically significant difference (*p* ≤ 0.05) is indicated by different superscript characters.

The data presented in Figure 4 revealed that the DLM-treated group displayed a drastic up-regulation of caspase3 expression alongside dramatic down-regulation of Bcl2 expression levels in hepatic and renal tissues when compared to the other groups. Interestingly, the DLM+NAC treated group showed remarkable down-regulation of hepatic and renal caspase3 and up-regulation of hepatic and renal Bcl2 when compared to the DLM group.

### 3.5. The Effect of DLM and/or NAC on Histopathological Character of Liver Tissues

**Figure 5:**
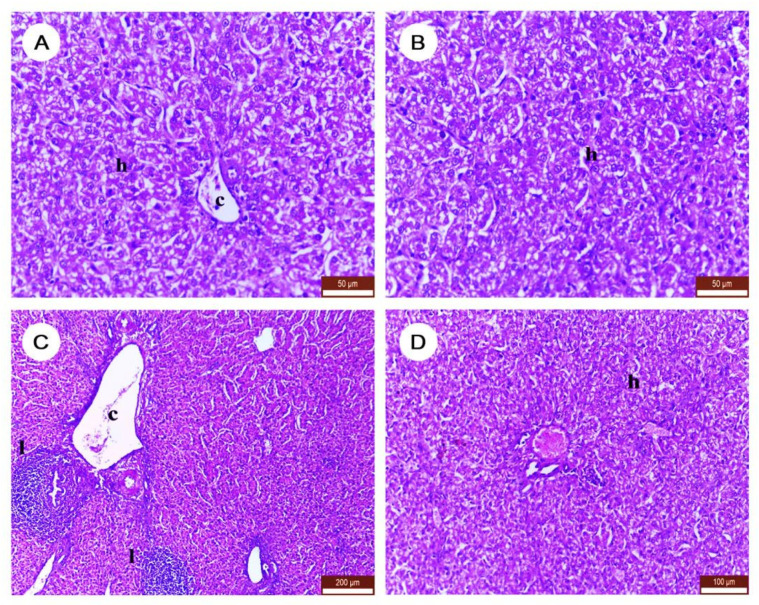
Changes in the liver histopathology after treatment with DLM and/or NAC. (**A**) Control group (scale bar = 50 µm), (**B**) NAC group (scale bar = 50 µm), (**C**) DLM (scale bar = 200 µm); and (**D**) DM+NAC (scale bar = 100 µm). c: Central vein, h: hepatic lobules, l: inflammatory cells infiltration. (H&E stain).

Expectedly, the histopathological examination of liver tissue obtained from control and NAC-treated chickens showed a normal histological structure of hepatic lobules, sinusoids, and portal area (Figure 5A,B). The DLM group exhibited alterations in the histological picture represented by widened portal blood vessels with congestion, inflammatory cells between the hepatic cords, and thickened wall of the hepatic blood vessels (Figure 5C). However, chickens co-administrated with both DLM and NAC showed lesser damages when compared to the DLM-exposed group (Figure 5D).

### 3.6. The Effect of DM and/or NAC on Histopathological Character of Kidney Tissues

**Figure 6:**
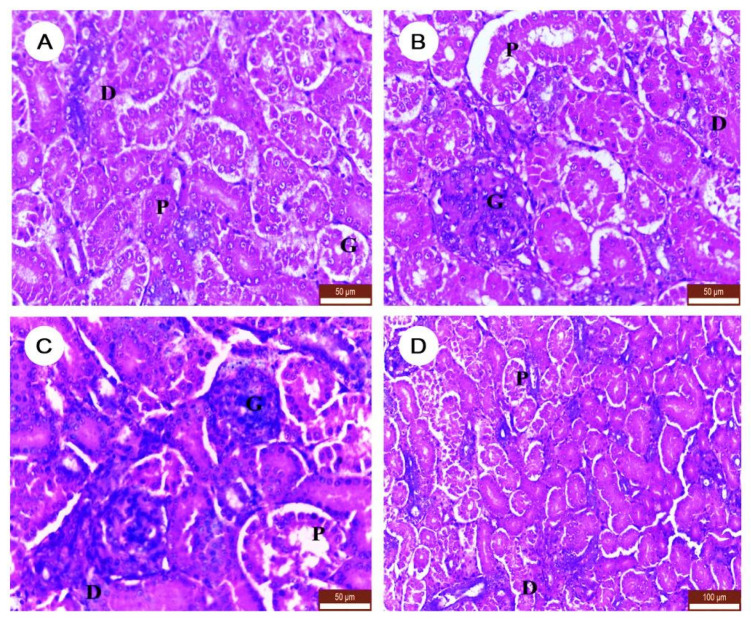
Changes in the kidney histopathology after treatment with DLM and/or NAC. (**A**) Control group (scale bar = 50 µm), (**B**) NAC group (scale bar = 50 µm), (**C**) DLM (scale bar = 50 µm); and (**D**) DM+NAC (scale bar = 100 µm). G: Glomerulus, P: proximal tubule, D: distal tubule (H&E stain).

Kidneys of the control and NAC groups showed normal architecture of the renal structure including renal corpuscles, proximal tubules, and distal tubules (Figure 6A,B). Contrarily, DLM-exposed birds caused severe renal injury indicated by hyperplasia and necrosis in renal glomeruli, tubular dilatation, sloughing of apical epithelium, and lymphocytic cell infiltration (Figure 6C). Observably, the co-treatment with NAC could attenuate the DLM-induced kidney injury seen by improvements in the forementioned lesions in comparison to DLM only treatment (Figure 6D).

## 4. Discussion

Environmental pollution is one of the leading causes of health problems [27,28]. DLM, a pyrethroid insecticide, is widely used in agriculture and veterinary medicine around the world, posing a risk of non-target organisms such as humans and farm animals being exposed [29].

In the current study, as expected, the DLM-treated group showed a significantly lower FCR comparing to the control, NAC, and DLM+NAC groups. This may be due to the pesticide’s anorectic properties combined with low feed-conversion quality. Excessive replication of saprophytic bacteria in poultry feed results in the release of various metabolites that can enhance the toxicity of DLM in terms of slowing growth rate [30]. Moreover, our data revealed a remarkable hepato-renal dysfunction after DLM exposure, indicated by elevated AST and ALT activities along with substantial increases in creatinine and urea levels. These data were in parallel to those obtained by Abdel-Daim et al. [31]. However, co-treatment with NAC markedly restored these markers close to normal levels in the DLM-intoxicated birds. This means that NAC could protect the liver and kidney from the DLM-induced damage by suppressing enzyme leakage across cellular membranes, preserving the integrity of plasma membranes, and thereby restoring the levels of these enzymes.

The oxidative stress caused by overproduction of free radical was blamed for the DLM-induced hepato-renal injuries [31]. Several researches documented that DLM may cause an increase in ROS production, resulting in initiation of oxidative damage in rat’s tissues such as the liver, kidney, and brain [4,31,32]. In addition, DLM has been reported to cause oxidative stress, resulting in body damage in the freshwater Nile tilapia [5,33].

It is well known that pesticide poisoning causes oxidative damage via a promoted generation of ROS and induction of lipid peroxidation [34]. ROS mediate the toxic impact of pyrethroid insecticides [35], and oxidative stress is the primary cause of liver harm [36,37]. MDA is a well-known by-product of lipid peroxidation and has been used as an oxidative stress marker [38,39]. Accumulative evidence suggested that a large amount of hydroxyl radical (OH^•^) are produced during oxidative stress. OH^•^ is the most injurious radical compared to other ROS that has a great affinity to the lipid content of the cell membrane [34,39,40,41,42,43]. Therefore, it is strongly suggested that the increased MDA levels was associated with DLM-induced ROS production.

In consistence with our previous studies, lipid peroxidation and loss of the cell membrane integrity are the main causes participating in the leakage of liver transaminases into the blood stream leading to elevation of their levels in the serum [34,39,41,42,43]. Moreover, the observed loss of the renal brush border in the histopathological examination might be attributed to the DLM-induced lipid peroxidation; where, the DLM-generated ROS disrupts the lipid content of the apical membrane [39,44]. Consequently, progressive accumulation of sloughed epithelia in the tubular lumen might explain the dilated tubules in the DLM-injured group [43]. It is strongly reported that inflammatory response is associated with oxidative stress; herein, the inflammatory cell infiltrations were seen in the liver and kidney tissue after DLM exposure [40,45]. All of these observed pathological lesions were improved after supplementation of NAC.

Reduced-glutathione (GSH) is a cellular antioxidant that plays a crucial role in the scavenging of ROS [40,43]. SOD is required for dismutation of the normally generated superoxide anion (O_2_^•−^). However, when the O_2_^•−^ is over generated, the SOD is overwhelmed leading to disruption of the cellular oxidant/antioxidant balance [40]. The present investigation revealed enhanced lipid peroxidation alongside dramatic reductions in SOD activity and GSH level, affirming that oxidative stress is a key factor in DLM-induced liver and kidney damage.

Thiol group content of GSH is the main modulator of GSH function in conjugation with the generated ROS. Therefore, NAC supplementation provides an alternative source of thiol group offering other avenues for ROS-conjugation which helps to restore the depleted cellular GSH [17]. Consistently, the present data indicated the potential use of NAC in regeneration GSH in the DLM-intoxicated chicken. These findings are consistent with Wong et al. [46], who found that NAC can regulate GSH levels and protect the liver against reactive metabolite damage in a rat model.

**Figure 7:**
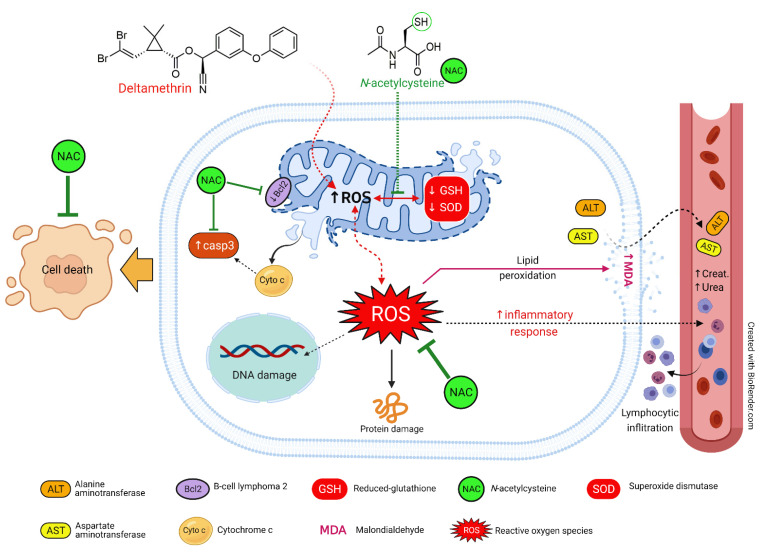
The proposed mechanistic insights associated with the protective activity of NAC against DLM-induced toxicity.

Accumulative evidence suggested that the progressive injury caused by uncontrolled ROS and oxidative damage will be ended by cell death due to stimulation of the apoptotic cascade [47]. Bcl2 is an antiapoptotic protein that is mainly involved in the intrinsic apoptotic pathway, also known as the mitochondria-associated pathway [48,49]. It is well documented that the mitochondrial membrane loses its integrity in oxidative stress; thereby, the cytochrome c is released into the cytoplasm, leading to up-regulation of the downstream apoptotic proteins caspases, including caspase3 along with up-regulation of the antiapoptotic proteins such as Bcl2 [49]. Our findings revealed the occurrence of apoptosis in DLM-intoxicated chickens, as evidenced by increased caspase3 and decreased Bcl2 mRNA expression levels in the liver and kidney. Khalaf and his group found that the insecticide increased the expression of the caspase3 [50], confirming our obtained data. Our previous studies with others have linked mitochondrial dysfunction to apoptosis through the development of ROS and the activation of caspases [24,34,39,40,41,51]. Interestingly, in the present investigation, treatment with the antioxidant NAC reversed DLM-induced apoptotic responses in hepatic and renal tissue, revealing the antiapoptotic competence of NAC against DLM-induced tissue injury. Figure 7 summarizes the mechanistic insights associated with the protective activity of NAC against DLM-induced toxicity.

## 5. Conclusions

In the present work, DLM caused dramatic oxidative damage in the liver and kidney tissue indicated by increased MDA concentration and exhaustion of the endogenous antioxidant system. However, the overall results confirm the protective effect of NAC in DLM-induced hepato-renal toxicity in broiler chicken by its ability to scavenge free radicals and regenerate the GSH, which helped restore the cellular redox hemostasis. We anticipate that NAC would a potential feed supplement for broiler chicken posing potent antioxidant activity against DLM or other pesticide contaminants.

## Figures and Tables

**Table 1:** The formulation of the basal diet for broilers.

Ingredients (kg/100 kg)	Starter Diet (0 to 21 Days)	Finisher Diet (22–35 Days)
Yellow corn	58.57	64
Soyabean meal (48%)	32	25
Corn gluten (60%)	5	5
Soybean oil	0.7	2.5
Di-calcium phosphate (22% Ca and 19% Ph)	1.5	0.95
Limestone (35% Ca)	1.7	1.74
Common salts	0.2	0.15
Methionine (95%)	0.13	0.36
Lysine (98%)	0	0.1
Vitamins and mineral premix *	0.2	0.2
**Calculated composition**		
Protein (%)	23	20
k. calory ME/kg	2950	3120
Calcium (%)	1	0.9
Phosphorus (%)	0.48	0.35

* Each 2 kg contain the following vitamins and minerals: Vit. A 12 MIU, Vit. D_3_ 2 MIU, Vit. E 1000 mg, Vit. k_3_ 1000 mg, Vit. B_1_ 1000 mg, Vit. B_2_ 5000 mg, Vit. B_6_ 1500 mg, Vit. B_12_ 10 mg, biotin 50 mg, pantothinic acid 10 g, nicotinic acid 30 g, folic acid 1000 mg, manganese 60 g, zinc 50 g, iron 30 g, copper 4 g, iodine 300 mg, selenium 100 mg, cobalt 100 mg, carrier (CaCO_3_) to 3 kg. (Vitamin-mineral premix for poultry, Agrivet Pharma, Cairo, Egypt).

**Table 2:** Primer pairs sequences of caspase3, Bcl2, and β-actin genes.

Gene Name	Primer Sequence (5′-3′)	Reference
*Caspase3*	F: TGGCCCTCTTGAACTGAAAG R: TCCACTGTCTGCTTCAATACC	[24]
*Bcl2*	F: ATCGTCGCCTTCTTCGAGTT R: ATCCCATCCTCCGTTGTTCT	[25]
*ß-actin*	F: CCACCGCAAATGCTTCTAAAC R: AAGACTGCTGCTGACACCTTC	[26]

**Table 3:** Effect of DLM and/or NAC on growth performance.

Parameters	Control	NAC	DLM	DLM+NAC
B. wt (7 days; g)	143.80 ± 0.50 ^a^	144.13 ± 0.97 ^a^	139.27 ± 0.41 ^b^	143.27 ± 1.01 ^a^
B. wt (35 days; g)	1938.6 ± 2.11 ^b^	2119.6 ± 1.73 ^a^	1526.7 ± 8.70 ^d^	1761.9 ± 8.92 ^c^
Weight gain (g)	1794.8 ± 2.28 ^b^	1975.5 ± 1.52 ^a^	1387.5 ± 8.76 ^d^	1618.6 ± 9.08 ^c^
Feed intake (g)	3254.3 ± 2.48 ^c^	3383.3 ± 7.17 ^a^	3931.7 ± 2.63 ^d^	3286.3 ± 13.10 ^b^
FCR (%)	1.81 ± 0.002 ^c^	1.71 ± 0.004 ^d^	2.11 ± 0.013 ^a^	2.03 ± 0.016 ^b^

All data are expressed means ± SE (*n* = 3). B. wt; body weight, FCR; feed conversion ratio. The means with different superscript letters are considerad statistically significant at *p* ≤ 0.05 in the same raw.

## Data Availability

Upon request, the data utilized to verify the findings of this research are obtainable from the corresponding authors.
